# Transposable element dynamics and regulatory role in sika deer (*Cervus nippon*) antler development

**DOI:** 10.1186/s13100-026-00411-1

**Published:** 2026-07-15

**Authors:** Qianghui Wang, Haihua Xing, Yukai Ma, Zihui Sun, Ruobing Han, Heping Li

**Affiliations:** 1 College of Wildlife and Protected Area, Northeast Forestry University, Harbin, 150040 China; 2 Key Laboratory of Forest Resources Conservation and Utilization in the Southwest Mountains of China, Ministry of Education, Key Laboratory for Conserving Wildlife With Small Populations in Yunnan, College of Forestry, Southwest Forestry University, Kunming, China

**Keywords:** Transposable elements, Sika deer, Genomics, TE expression, Antler development

## Abstract

**Background:**

Deer antlers represent one of the few mammalian organs capable of complete regeneration, yet the underlying genomic regulatory mechanisms remain incompletely understood. Transposable elements (TEs), as key drivers of genome evolution and gene regulation, may play pivotal roles in the evolution of complex traits. This study aimed to systematically investigate the evolutionary dynamics of TEs in sika deer genome and to elucidate their potential regulatory functions in the development of antler by integrating comparative and functional genomics approaches.

**Results:**

Comparative genomics revealed a lineage-specific massive expansion of LINE retrotransposons (dominated by RTE-BovB and L1 subfamilies) in cervid genomes, particularly in sika deer and red deer, where they constitute over 29% of the genomic sequence. The peak of this expansion (~ 7.5 million years ago) coincides with the major cladogenesis events within *Cervus*. Expression analysis showed that approximately 8.88% of annotated TEs are transcriptionally active in antler mesenchyme, with LINE and LTR elements being predominant among the 1,191 TEs consistently highly expressed across all stages. We identified numerous stage-specifically expressed TEs (SETEs) and found positive correlations between SETEs and key antler development genes, such as the osteogenic master regulator *RUNX2*, the extracellular matrix remodeling gene *ANPEP*, and the collagen synthesis gene *P4HA3*. Enrichment analysis demonstrated that genes adjacent to these TEs are significantly involved in pathways crucial for regeneration and rapid growth, including Wnt signaling, Hippo signaling, regulation of the actin cytoskeleton, and protein digestion and absorption.

**Conclusions:**

Our findings point to a potential role for cervid TEs expansion in driving genomic evolution and the acquisition of cervid-specific phenotypes, particularly antler growth and development. Through profiling TE expression in antler mesenchymal tissue across key development stages, the results suggest that dynamic TEs expression may be involved in the regulation of antler development, implicating a potential regulatory role for TEs in this rapid growth process. This study provides new insights into the molecular mechanisms underlying the rapid growth of antlers.

**Supplementary Information:**

The online version contains supplementary material available at 10.1186/s13100-026-00411-1.

## Background

The sika deer (*Cervus nippon*) represents a pivotal genetic resource within the Cervidae family, exhibits an annual cycle of antler pedicle shedding and regeneration in males [1, 2]. This remarkable biological characteristic makes antler the only mammalian organ with periodic complete regenerative capacity [3, 4]. Antler distinctive growth pattern demonstrated through rapid growth, temporally coordinated tissue differentiation, and systematic ossification constitutes a compelling model for biological investigation [5–9]. The growth of deer antlers initiates a rapid proliferation phase at 30 days (the small-saddle stage), attains its peak growth rate at 60 days (the two-branched stage), and subsequently transitions into the ossification phase by the three-branched stage (after 90 days). Consequently, these three temporal nodes are widely considered in the literature as critical stages in the antler growth and developmental process [4, 6–8]. The study of antler regeneration not only illuminates fundamental principles of mammalian organ regeneration but also presents critical opportunities for translational applications in regenerative medicine and developmental biology.

Transposable elements (TEs) are mobile, repetitive, and diverse segments of DNA which occupy large portions of eukaryotic genomes, including more than half of the human genome [10, 11]. TEs were initially viewed as selfish DNA or parasitic elements because of their deleterious effects on host genomes [12, 13]. However, recent studies have demonstrated that TE play important roles in driving the evolution of genomes and transcriptional regulation [14, 15]. They can function as cis-regulatory elements—such as enhancers, promoters, and insulators—that influence the expression of nearby genes [15, 16]. Furthermore, through exaptation, TEs can be co-opted into functional genomic elements that drive evolutionary adaptations [17]. In various mammalian species, TEs have been shown to play pivotal roles in key developmental processes, including embryogenesis, placental development, and tissue-specific differentiation [11, 15, 16]. Their ability to reshape gene regulatory networks positions TEs as potential key players in the evolution of complex traits [17, 18], including extraordinary biological phenomena like antler regeneration and development.

Recent advances in genomic technologies, particularly next-generation sequencing (NGS), have enabled comprehensive multi-omics investigations into antler biology. Pioneering work by research groups such as those led by Chen and Qin has systematically characterized the genomic and cellular basis of antler regeneration and development, from comparative genomics across Artiodactyla to single-cell transcriptomics of sika deer [3, 5]. Complementary studies by Li, Xing, and others have generated extensive transcriptomic data, greatly enriching our understanding of the molecular dynamics during antler growth. These efforts have identified critical genes, signaling pathways, and cellular processes underlying antler development [2, 7, 8, 19].

However, despite these significant advances, the potential involvement of TEs in regulating antler development remains largely unexplored. A systematic analysis of TE distribution, evolutionary dynamics, and regulatory functions in cervid genomes is notably absent from the current literature. This gap is particularly striking given the extraordinary development capacity of antlers and the established role of TEs in driving regulatory innovation in other biological contexts.

This study aims to address this knowledge gap by conducting a comparative analysis of TE distributions across the sika deer genome and related species including cattle (*Bos taurus*), sheep (*Ovis aries‌*), pig (*Sus scrofa*), red deer (*Cervus elaphus*), and reindeer (*Rangifer tarandus platyrhyncus*). We aim to identify cervid-specific TE patterns that may have contributed to the evolution of antler development. Furthermore, we will investigate potential regulatory roles of TEs in key genes and pathways involved in the rapid growth of antlers. By integrating TE biology with antler growth and developmental genetics, this research promises to provide novel insights into the regulatory mechanisms governing antler growth and development, while simultaneously contributing to our broader understanding of mammalian organ regeneration and developmental plasticity.

## Results

### Systematic annotation and comparative analysis of transposable elements

**Fig. 1:**
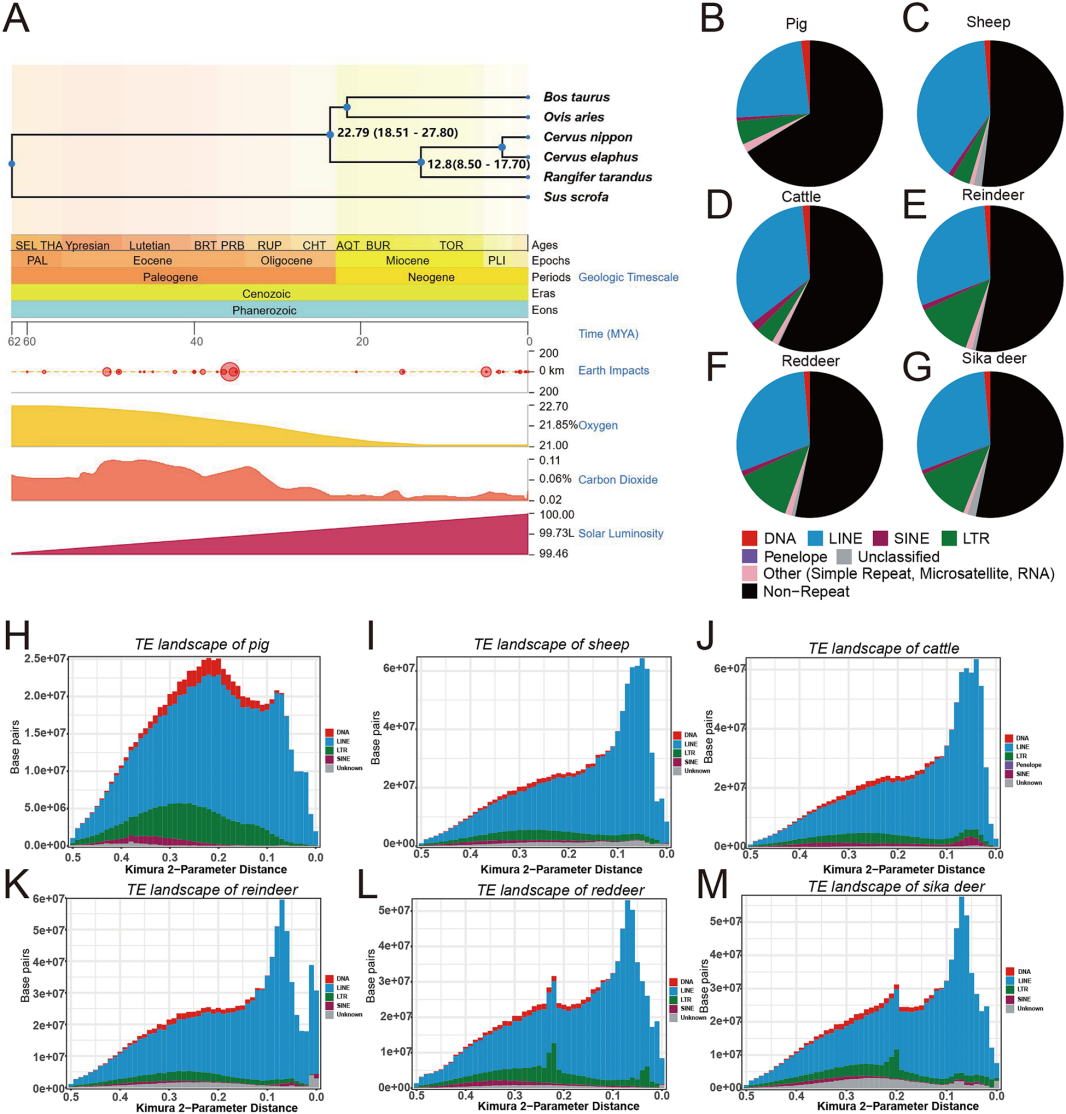
Landscape of TEs distribution in the genomes. **A** Phylogenetic relationships and estimated divergence times of six artiodactyl species; (**B**-**G**) Proportion of repetitive sequences in the different species genome; (**H**-**M**) TE distribution landscape in the different species genome

A comprehensive annotation and comparative analysis of TEs was performed across the genomes of 6 artiodactyl species (Fig. 1A). The results demonstrated different in the proportion of the differences genome occupied by TEs (Supplementary Table 1). Among they, the pig genome was the least dominated by repeats, with TEs accounting for approximately 32.11% of its sequence (Fig. 1B). The sheep genomes displayed the highest TE proportions of ~ 47.16% (Fig. 1C). The red deer and sika deer genomes exhibited relatively high TE content, comprising approximately 45.39% and 44.03% of their respective assemblies (Fig. 1F and G). The cattle and reindeer genome showed a slightly lower TEs proportions of ~ 42.79% and ~ 42.05%, respectively (Fig. 1D and E).

Regarding the physical dimensions of TE invasions, which are implicated in various biological processes such as speciation and the emergence of species-specific traits [20], our analysis revealed that the cumulative length of annotated TEs was exceeded 1.22 Gb in the sika deer genome. The other cervid and ruminant genomes (red deer, reindeer, cattle, sheep) also contained massive TE landscapes, with cumulative sizes ranging from 1.1 to 1.34 Gb. The pig genome, while having the lowest proportion, still contained a substantial ~ 0.8 Gb of annotated TEs. Additional, cervid genomes, particularly sika deer and red deer, were characterized by a remarkable expansion of LINE elements, which constituted over 29% of their genomes. A notable finding was the recent activity and abundance of cervid-specific SINE families in the deer species (Fig. 1K, L, and M). In the genome of cattle and sheep, the SINE represented a more substantial fraction compared to the other species (Fig. 1I and J). The pig genome displayed a more balanced distribution of LINE, SINE, and DNA transposon elements, with no single order exhibiting a dramatic expansion comparable to that seen in the cervids (Fig. 1H). DNA transposons were found to be relatively scarce in all analyzed artiodactyl genomes, typically comprising less than 2% of the genomic sequence.

In summary, this comparative analysis delineates the diverse genomic landscapes shaped by TEs in artiodactyls. The cervid genomes, particularly sika deer and red deer, harbor the most repeat-rich environments, largely driven by the proliferation of LINEs and cervid-specific SINEs. Ruminants like cattle and sheep show a significant influence of LTR retrotransposons, while the pig genome presents a less repetitive and more balanced TE profile. These differences underscore the dynamic and lineage-specific nature of TE evolution within the Artiodactyla order.

### Analysis of TE insertion time in sika deer

**Fig. 2:**
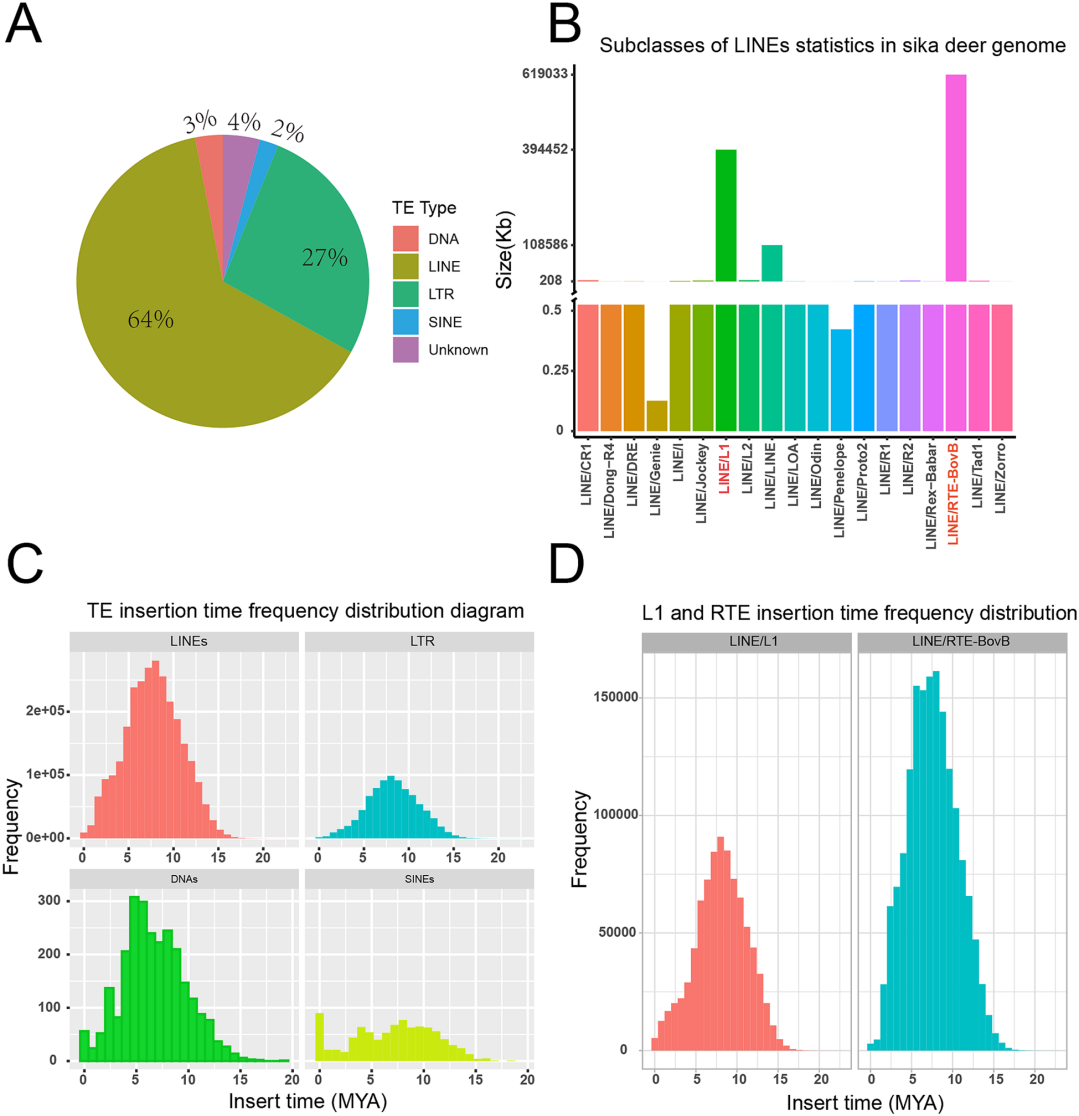
Proportion and insertion time of TEs in the sika deer genome. **A** Pie chart showing the proportion of different TE types; (**B**) Histogram illustrating the distribution of various subfamilies within LINE elements; (**C**) Frequency distribution of TE insertion time in the sika deer genome; (**D**) Frequency distribution of insertion time for L1 and RTE subclass

The insertion and expansion of TEs are frequently associated with genomic evolution and the development of species-specific characteristics [20–22]. Our analysis of TE composition in the sika deer genome (Fig. 2A) revealed that LINEs represent the predominant category, constituting 64% of all TEs, followed by LTRs (27%), Unknown (4%), DNA (3%), and SINEs (2%). Further statistical examination of the major LINE subfamilies (Fig. 2B) showed that LINE/RTE was the most abundant, followed by LINE/L1, with both subfamilies exceeding 100 Mb in cumulative length.

To further elucidate the evolutionary dynamics of TEs in sika deer, we estimated the insertion timing of different TE types (Fig. 2C). The results demonstrate that LINEs underwent a major expansion between 5 and 12 million years ago (Mya), with a pronounced peak at approximately 7.5 Mya. Similarly, LTR elements exhibited a significant burst of activity between 6 and 11 Mya, peaking around 8 Mya. In contrast, DNA transposons and SINEs showed relatively uniform insertion profiles over time, although SINEs displayed comparatively recent activity. Notably, a focused analysis of the two dominant LINE subfamilies, L1 and RTE, revealed that their peak insertion times aligned closely with the overall LINE expansion, also centering around 7.5 Mya (Fig. 2D).

### Expression of TEs in antler mesenchymal

**Fig. 3:**
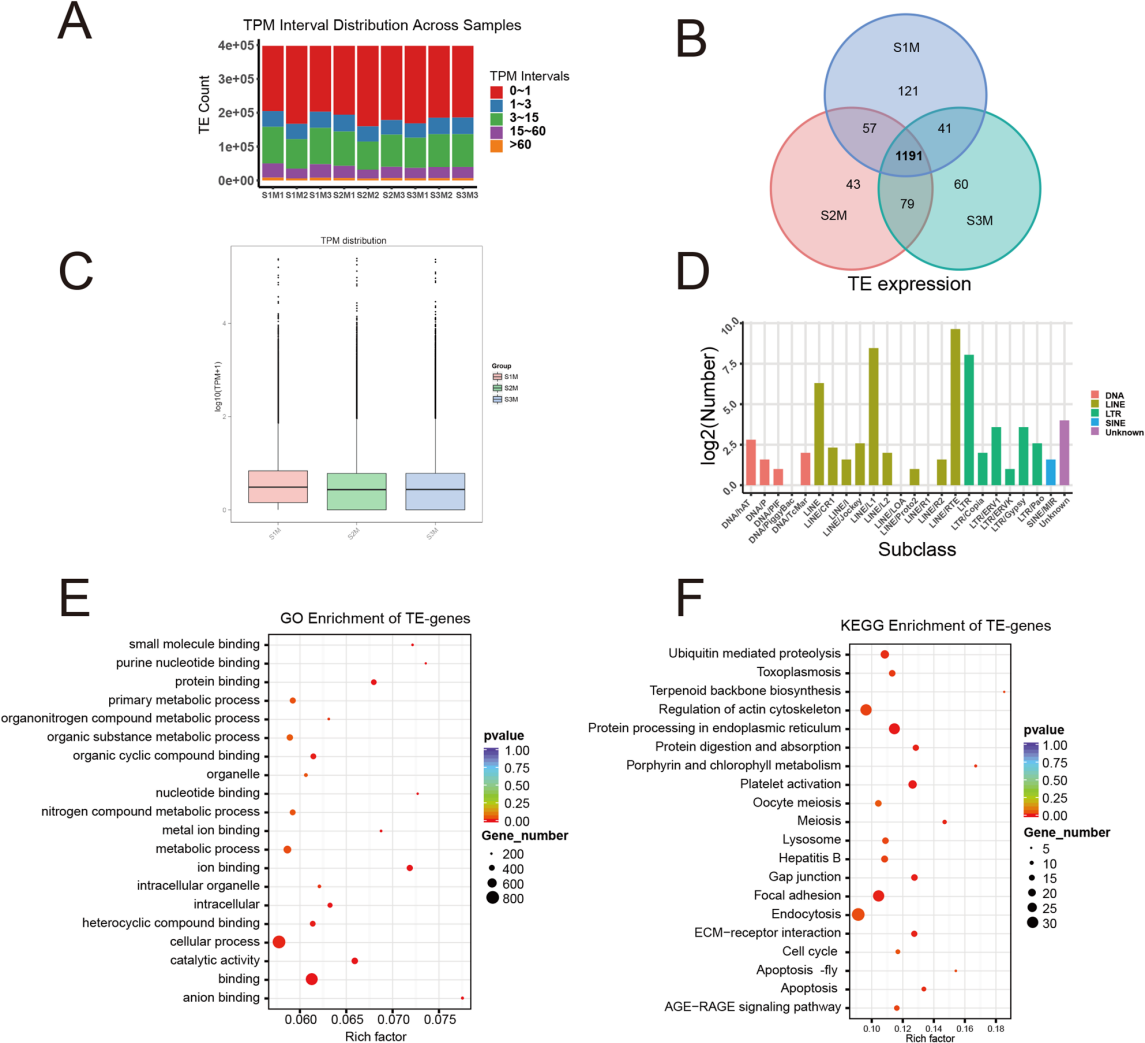
Expression of TEs in the antler mesenchyme of sika deer. **A** Distribution of TE expression levels; (**B**) Venn diagram illustrating the distribution of highly expressed TEs in the mesenchyme during the three key developmental stages; (**C**) Box plot comparing TE expression levels across three key developmental stages of antler growth; (**D**) Histogram showing the quantitative distribution of shared highly expressed TE subclass across the three developmental stages; (**E**) GO enrichment results of genes associated with highly expressed TEs; (**F**) KEGG enrichment results of genes associated with highly expressed TEs

The antler growth center is located in its tip mesenchyme [23]. To gain a deeper understanding of TEs expression patterns in antler of sika deer, we performed expression analysis on 4,477,139 annotated TEs in sika deer. Among these, 397,624 (~ 8.88%) TEs were found to be expressed (TPM > 1) in deer antler mesenchymal tissue (Fig. 3A, Supplementary Table 3). The distribution of expression levels revealed that the majority of TEs exhibited low expression levels (0–1), followed by those in the 3–15 range. TEs with expression levels between 1–3 and 15–60 was relatively common, while those exceeding 60 were the least abundant (Fig. 3A and B).

To further explore the key roles of TEs in antler growth and development, we characterized the TEs that were highly expressed in antler mesenchymal tissue during its growth at 30, 60, and 90 days. A total of 1,191 TEs were consistently highly expressed across these stages (Fig. 3C). Among the highly expressed TEs, LINEs were the most abundant, accounting for 78.83%, followed by LTR elements at approximately 18.9% (Fig. 3D). Furthermore, we investigated the genes located within 2 kb upstream and downstream of the highly expressed TEs. Functional enrichment analysis revealed that these genes are significantly involved in fundamental processes such as ion binding (*p* = 9.7e-12), cell cycle process (*p* = 0.003), transferase activity (*p* = 2.57e-9), collagen trimer (*p* = 3.07e-9), protein tyrosine kinase activity(*p* = 1.18e-6) and nitrogen compound metabolic process (*p* = 0.04), as well as broader metabolic regulation (Fig. 3E, Supplementary Table 5). KEGG pathway analysis further highlighted their roles in crucial pathways including Regulation of actin cytoskeleton (*p* = 0.025), Protein digestion and absorption (*p* = 0.005), Apoptosis (*p* = 0.01), and Focal adhesion (*p* = 0.004) (Fig. 3F, Supplementary Table 6). These findings suggest that TEs may play important regulatory roles in antler growth and development through influencing key molecular and cellular processes.

### Regulatory role of TEs in antler development

**Fig. 4:**
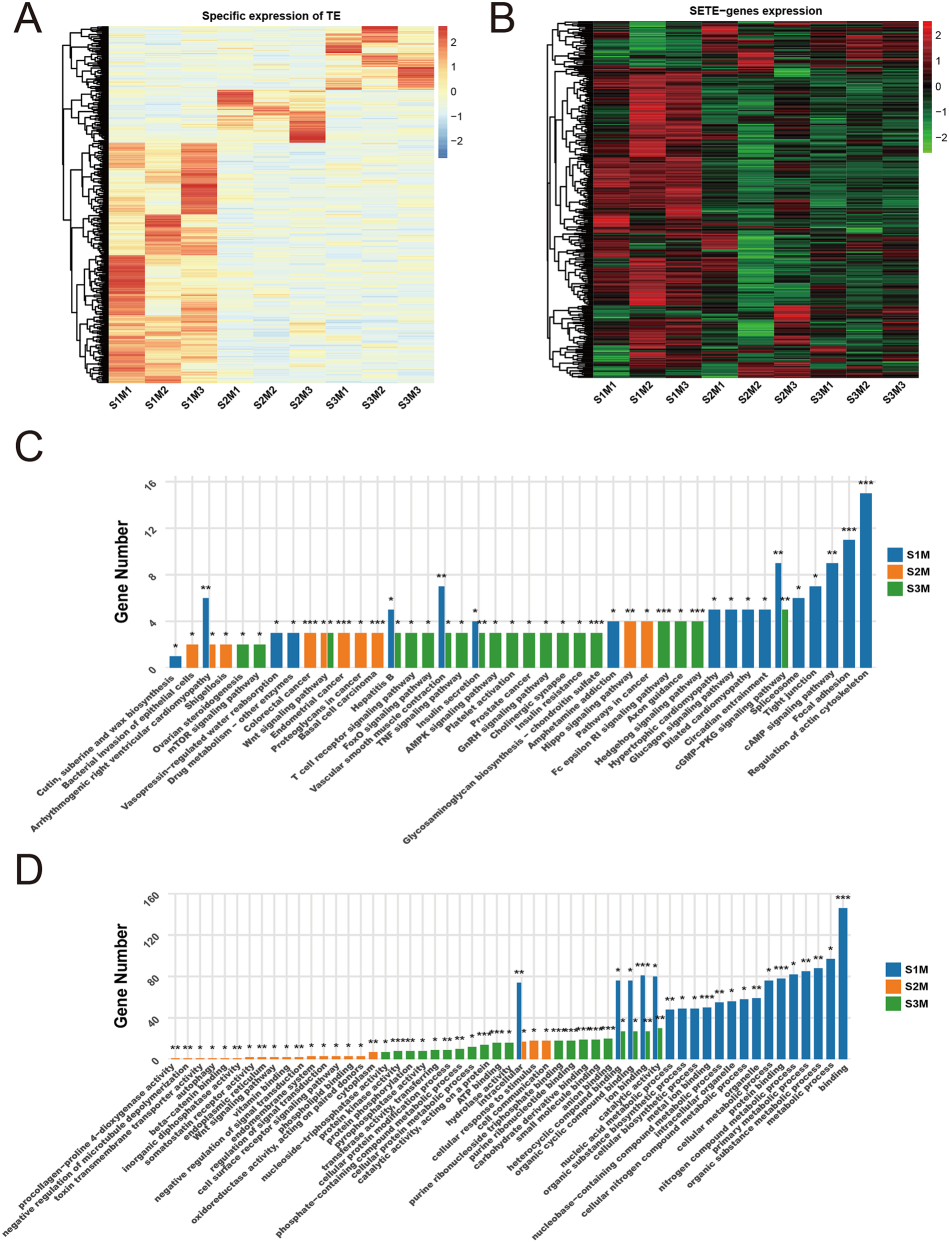
Expression of stage-specific TEs and their associated genes. **A** heatmap of SETEs expression; (**B**) Heatmap of gene expression associated with SETEs; (**C**) GO enrichment results of genes associated with SETEs; (**D**) KEGG enrichment results of genes associated with SETEs

To further elucidate the potential regulatory roles of TEs in antler growth and development, we identified stage-specifically expressed TEs during its growth at days 30, 60, and 90 days using the established method by She et al. [39]. This analysis revealed 295, 65, and 78 specifically expressed TEs in S1M, S2M, and S3M, respectively (Fig. 4A). We annotated genes flanking these TEs (Fig. 4B) and evaluated their expression relationships by calculating Pearson correlation coefficients. This identified 88, 5, and 2 significantly correlated TE–gene pairs in S1M, S2M, and S3M, respectively.In S1M, LINE/RTE-BovB family TEs showed strong correlations with several key genes, including TE0287920 with *P4HA3* (*r* = 0.80, FDR < 0.05), TE2755735 with Rab30 (*r* = 0.89, FDR < 0.05), TE1086902 with *ANPEP* (*r* = 0.93, FDR < 0.05), TE0930645 with *PARP8* (*r* = 0.94, FDR < 0.05), and TE3664199 with *KLHL4* (*r* = 0.94, FDR < 0.05). LTR-type TEs were also significantly correlated with genes such as TE0335209 with *Morf4l1* (*r* = 0.91, FDR < 0.05) and TE3580323 with *SRPX* (*r* = 0.83, FDR < 0.05). In S2M, notable correlations included TE0493160 with *VPS13B* (*r* = 0.99, FDR < 0.05), TE2458988 with *RUNX2* (*r* = 0.71, FDR < 0.05), TE3628761 with *OPHN1* (*r* = 0.99, FDR < 0.05), and TE2828231 with *IGF2* (*r* = 0.83, FDR < 0.05). In S3M, significant TE–gene correlations were observed for TE2897466 with *Rps6* (*r* = 0.99, FDR < 0.05) and TE5204203 with *Kcnma1* (*r* = 0.72, FDR < 0.05).

Functional enrichment analysis of genes associated with stage-specifically TEs revealed significant involvement in biological processes such as nitrogen compound metabolic process (*p* = 0.01), positive regulation of mitotic metaphase/anaphase transition (*p* = 0.0029), transferase activity, metal ion binding (*p* = 0.00017), positive regulation of apoptotic process (*p* = 0.019), negative regulation of signal transduction (*p* = 0.005), negative regulation of cell population proliferation (*p* = 0.0058), and negative regulation of microtubule depolymerization (*p* = 0.0058) (Fig. 4C, Supplementary Table 6–11). KEGG signaling pathways enriched among these genes included TNF signaling pathway(*p* = 0.015), Insulin secretion (*p* = 0.017), FoxO signaling pathway (*p* = 0.032), AMPK signaling pathway (*p* = 0.030), Wnt signaling pathway (*p* = 0.001), GnRH signaling pathway (*p* = 0.016), Insulin resistance (*p* = 0.016), mTOR signaling pathway (*p* = 0.04), Basal cell carcinoma (*p* = 0.0004), Regulation of actin cytoskeleton (*p* = 2.93e-06), and Hippo signaling pathways (*p* = 0.0017) (Fig. 4D, Supplementary Table 6–11).

## Discussion

The genomic architecture of mammals is not a static blueprint but a dynamic palimpsest, repeatedly rewritten by TEs [20–22]. Comparative genomics analysis across six artiodactyls has revealed a lineage-specific amplification of LINE retrotransposons in the sika deer (Fig. 1). Repetitive elements account for approximately 45.12% of the sika deer genome, with LINEs comprising 29.81% and LTRs accounting for 8.75%. While transposable element expansion is a relatively common phenomenon in mammalian evolution [24], the magnitude of LINE amplification in Cervidae warrants further comparative evaluation across the order. For context, the human genome contains LINEs at 20.4% and LTRs at 8.3%, while deer mouse (*Peromyscus maniculatus*) carries LINEs at 10% and LTRs at 11% [25]. This suggests that the Cervidae lineage may harbor a distinctive pattern of retrotransposon accumulation. TEs are known to provide cis-regulatory sequences and regulatory RNAs that influence gene expression at both transcriptional and post-transcriptional levels [15–17]. Recent studies have identified four transcription factors—MYC, KLF4, NFE2L2, and JDP2—with high regulatory activity during antler regeneration and development [26]. Notably, MYC expression appears to be driven by cervid-specific regulatory elements, which may contribute to the remarkable regenerative and growth capacity of antlers [26]. The expansion of LINE elements could have contributed regulatory potential that was co-opted for antler development and regeneration programs. However, the precise mechanistic connections between TE-derived regulatory sequences and antler regeneration pathways require further experimental validation.

The sheer dominance of LINE elements (particularly RTE-BovB and L1) in sika deer genomes, constituting over 29% of the sequence, represents an extreme deviation from the genomic landscapes of their artiodactyl relatives. This aligns with the burst model of TE evolution, where specific lineages undergo rapid expansions that can drive speciation and phenotypic diversification [27]. The temporal coincidence between the peak insertion of these LINEs (~ 7.5 Mya) and the major cladogenesis events within Cervidae is highly suggestive (Fig. 1A, Fig. 2C and D). This period of genomic turmoil likely generated extensive structural variation and epigenetic reshaping, creating a unique genomic context for evolutionary innovation. In contrast, the more balanced TE profile in pig and the LTR-dominated landscape in cattle, and sheep underscore the order-specific trajectories of genome evolution, supporting the notion that TE activity is a major force in shaping lineage-specific biology [28].

Crucially, the legacy of this expansion is not fossilized but functionally active. The significant expression of TEs (~ 8.88%) in antler mesenchyme—a site of epimorphic regeneration with parallels to blastema formation—indicates a role beyond selfish propagation. This echoes findings in other regenerative models, where TE-derived sequences are often co-opted as regulatory modules [29]. The significant overrepresentation of LINEs and LTR elements among TEs consistently highly expressed across developmental stages strongly implies their functional integration into transcriptional networks. The stage-specifically expression patterns and the discovery of highly correlated TE-gene pairs provide a new insight for antler growth molecular mechanism.

Notably, the significance correlations between SETEs and key developmental genes offer a new insight for TE-mediated regulation. For instance, TE2458988 maybe modulates antler ossification via co-expression with *RUNX2*. *RUNX2* activity is central to antler ossification, and its regulation by distal enhancers is well-established [30]. Similarly, correlations with *ANPEP* (involved in matrix remodeling) and *P4HA3* (collagen maturation) directly link TE activity to the intense extracellular matrix synthesis and turnover that characterizes antler growth [31]. TE0335209 association with *Morf4l1*, a gene involved in chromatin remodeling, further suggests a deeper, epigenetic layer of regulation, where TEs might influence local chromatin states to modulate developmental gene batteries [32]. We acknowledge the possibility that observed correlations between TEs and developmental genes may partially reflect passive transcription of TEs residing in open chromatin regions. This is a valid concern, as co-localization in transcriptionally active regions can generate spurious associations without direct regulatory interaction. Several studies have documented that TEs within open chromatin frequently show expression patterns that correlate with nearby genes due to shared transcriptional environments rather than direct regulatory relationships [33]. This possibility warrants rigorous experimental validation in future studies.

The functional enrichment results further supported this view. The significant enrichment of TE related genes in pathways like Wnt signaling, Hippo signaling, and Regulation of actin cytoskeleton is not coincidental. These pathways are fundamental hubs governing cell fate determination, proliferation, and tissue patterning—processes paramount to regeneration [34]. The concurrent enrichment in protein digestion and absorption and focal adhesion underscores the tailored adaptation for rapid, scaffold-based growth. This pattern suggests that the cervid-specific TE invasion did not randomly affect the genome but was preferentially co-opted into networks central to antler growth and development. This is consistent with the regulatory network exaptation hypothesis, where TEs donate transcription factor binding sites to pre-existing gene networks, potentiating or modifying their output [35].

Therefore, we propose a refined model: The mid-Miocene burst of LINE retrotransposition in the cervid ancestor created a vast array of new, mostly neutral sequences. During the evolution of antlers—a trait likely under intense sexual and natural selection—a subset of these TE insertions, fortuitously located near genes in pathways critical for rapid bone growth and regeneration (e.g., Wnt, Hippo, cytoskeletal organization), were functionally harnessed. They evolved into stage-specifically regulatory elements, adding new nodes and layers of control to the ancestral mammalian limb development program. This exaptation event effectively rewired the genomic regulatory circuitry, enhancing the speed, scale, and cyclic nature of antler growth. This model positions TEs not as junk, but as key facilitators of macroevolutionary change, providing the *cis*-regulatory diversity necessary for the emergence of complex, novel traits [36].

## Conclusions

This study elucidates a potential link between genomic dynamics and an iconic phenotypic innovation. We found that the exceptional expansion of LINEs, specifically the RTE-BovB and L1 subfamilies, is a genomic signature of the cervid lineage, temporally coupled with its diversification. From TEs active transcription in the antler mesenchyme to their stage-specifically co-expression with master regulators of osteogenesis and key signaling pathways, these TEs appear embedded in the antler's developmental program.

Our findings support the hypothesis that the cervid-specific TE invasion supplied a critical substrate of evolutionary potential. The subsequent exaptation of these elements into novel *cis*-regulatory components likely facilitated the evolution of the antler’s unparalleled regenerative capacity by augmenting and refining gene regulatory networks controlling growth, metabolism, and patterning. This work underscores the transformative power of TEs in driving phenotypic evolution. Future research employing functional genomics (e.g., chromatin conformation capture, CRISPR-mediated deletion of specific TEs) will be essential to definitively validate the enhancer functions proposed here and to fully decipher the regulatory grammar written by TEs in this phenomenon.

## Methods

### Performing systematic TE annotation and comparison

To systematically compare the distribution patterns and explore the potential functions of TEs in the sika deer genome, as well as their possible role in antler development, we retrieved the latest genome assemblies of 5 species — pig (GCF_000003025.6), cattle (GCA_002263795.4), sheep (GCA_040805955.1‌), reindeer (GCA_949782905.1), and red deer (GCF_910594005.1) — from the NCBI public database. Together with the high-quality sika deer (GCA_040085125.1) genome assembled in our laboratory [9], the 6 genomes were subjected to systematic annotation. EarlGrey (v5.1.0) [37] with the uniform parameter set: -t 16 -r eukarya -f 1000 -m no -n 20 was employed to systematic annotation for repetitive sequence identification and classification. This step generated annotated repetitive sequences for each species. From the output, we filtered and identified TEs in each genome and converted the results into standard GFF3 format files for downstream analyses. To contextualize TE evolutionary dynamics within a phylogenetic framework, we further obtained the phylogenetic relationships and estimated divergence times of the 6 species from the TimeTree database (http://www.timetree.org/), as illustrated in Fig. 1A.

### Analysis of TEs insertion timing

To investigate the potential association between antler development and TEs, we conducted a systematic analysis of the relative abundance and insertion chronology of various TE types in the sika deer genome. The synonymous substitution (dS) rate was estimated using a free-ratio model implemented in the codeml script in PAML (v4.8) [38] based on the protein-coding sequences of the sika deer genome, and dS was used as the nucleotide substitution rate in the evolutionary process of sika deer sequences. Divergences of TEs from the consensus sequences extracted from EarlGrey analysis results were adjusted for multiple substitutions using the Jukes-Cantor formula K = −300/4 × Ln(1-D × 4/300), where D represents the distance between the fragmented repeat and the consensus sequence. Insertion times of TEs were estimated using the equation T = K/2r [39], where T is the insertion time, and r is the nucleotide substitution rate for sika deer.

### TEs expression analysis in antler mesenchyme

We leveraged previously generated transcriptomic data from our laboratory to investigate TE expression in antlers (Supplementary Table 2) [40]. This dataset comprised 9 sika deer antler samples collected at three key developmental stages: 30, 60, and 90 days. TE expression levels were quantified using salmon (v1.10.3) with default parameters, following the method described by Kong et al. [41]. Firstly, we constructed a salmon index directly from TE sequences rather than the whole genome. Specifically, we extracted all annotated TE sequences from the sika deer genome and compiled them into a FASTA file. Then, salmon employs a selective‑alignment algorithm that uses fast‑to‑compute proxies to determine the potential mapping loci of each read, then scores and validates these mappings using a dynamic programming algorithm. For reads that map to multiple TE families with similar scores, salmon applies an expectation‑maximization (EM) algorithm that probabilistically distributes multi‑mapping reads among candidate TE sequences based on their relative abundances. Finally, expression level was reported in transcripts per million (TPM). For each developmental stage, the mean TPM value across the three biological replicates was calculated as the representative expression level of each TE. TEs with expression levels three-fold higher than the mean expression of all expressed TEs in a given stage were defined as stage highly expressed TEs. To explore the potential functions of TEs commonly highly expressed across all three stages, we extracted genes located within 2 kb upstream and downstream of these TEs and performed Kyoto Encyclopedia of Genes and Genomes (KEGG) and Gene Ontology (GO) enrichment analyses using the EnrichmentPipeline [42]. For GO enrichment, we used the clusterProfiler package (v4.0) in R, which implements a hypergeometric test to identify GO terms that are overrepresented among the query gene set relative to the background genome. GO annotations for sika deer genes were obtained from the Ensembl database via the biomaRt package, and enrichment was performed separately for the three GO ontologies: biological process (BP), cellular component (CC), and molecular function (MF). For KEGG pathway enrichment, we employed the same hypergeometric test implemented in clusterProfiler, using KEGG orthology (KO) assignments derived from KAAS (KEGG Automatic Annotation Server) to map sika deer genes to their corresponding KEGG pathways. In both cases, a false discovery rate (FDR) correction was applied using the Benjamini–Hochberg method to adjust for multiple testing, and significantly enriched terms were defined as those with an adjusted *p*-value < 0.05.

### Specific expression patterns of TEs

To further research the function of TEs in antler different development stage, the stringent standards as previously described [43] was used to detect specific expression TEs (SETEs). In brief, SETEs were defined that the TEs with expression level (TPM) in the different stage according to criteria: (1) the TPM value of the candidate TEs in one stage was more than three times that of other stage, (2) the TPM value of candidate TEs target stage was greater than 50% of the average expression level in all other stages, and (3) the expression level of candidate TEs was at the top 25% of all TEs in each stage. Additionally, bedtools (v2-2.25.0) was applied to extract SETE-genes, which located within 2 kb upstream and downstream in SETEs. KEGG and GO enrichment analyses for the SETE-genes were performed using the EnrichmentPipeline [42].

### Investigating TE-Genes expression interactions for stage-specific TEs

To assess whether TEs may influence the expression of key genes during antler development, we quantified gene expression levels across the three key developmental stages. Using the previously published sika deer genome as a reference [9]. high‑quality sequencing reads from each sample were mapped to the genome with HISAT2 (v2.0.4) [44]. Read counts per gene were obtained using HTSeq (v0.6.0) [45], and gene expression levels were calculated in fragments per kilobase of transcript per million mapped fragments (FPKM) according to the formula: FPKM = (number of reads mapped to a gene × 10⁹)/(total reads mapped to genes × gene length in bases). Pearson correlation coefficients between the expression levels of SETEs and their associated genes were then computed using the stats module from the SciPy package in Python to identify TE‑gene pairs with statistically significant associations, and FDR correction was applied using the Benjamini–Hochberg method to adjust.

### Visualization

In this study, with the exception of Fig. 1A, all other figures were generated using R (v4.5.0), primarily employing the ggplot2 and pheatmap packages.

## Supplementary Information

Supplementary Material 1: Supplementary Table 1：Statistical results of repeat sequence identification and analysis. Supplementary Table 2: RNA-seq data quality and statistical results. Supplementary Table 3: TEs expression levels (TPM) in antler mesenchyme. Supplementary Table 4：The GO enrichment results of highly expressed TE related gene. Supplementary Table 5：The KEGG enrichment results of highly expressed TE related gene. Supplementary Table 6：The GO enrichment results of SETE-gene in S1M. Supplementary Table 7：The KEGG enrichment results of SETE-gene in S1M. Supplementary Table 8：The GO enrichment results of SETE-gene in S2M. Supplementary Table 9：The KEGG enrichment results of SETE-gene in S2M. Supplementary Table 10：The GO enrichment results of SETE-gene in S3M. Supplementary Table 11：The KEGG enrichment results of SETE-gene in S3M.

## Data Availability

All raw sequencing data are from publicly released databases and are accessible via download links available in the cited literature. Meanwhile, the data that support the findings of this study are available from the corresponding author upon reasonable request.

## References

[1] Han R, Han L, Zhao X, Wang Q, et al. Haplotype-resolved genome of sika deer reveals allele-specific gene expression and chromosome evolution. Genom Proteom Bioinform. 2023;21(3):470–82. 10.1016/j.gpb.2022.11.001 PMC10787017 36395998

[2] Xing X, Ai C, Wang T, Li Y, et al. The first high-quality reference genome of sika deer provides insights into high-tannin adaptation. Genom Proteom Bioinform. 2023;21(1):203–15. 10.1016/j.gpb.2022.05.008 PMC10372904 35718271

[3] Qin T, Zhang G, Zheng Y, et al. A population of stem cells with strong regenerative potential discovered in deer antlers. Science. 2023;379(6634):840–7. 36821675 10.1126/science.add0488

[4] Li C, Zhao H, Liu Z, McMahon C. Deer antler-a novel model for studying organ regeneration in mammals. Int J Biochem Cell Biol. 2014;56:111–22. 25046387 10.1016/j.biocel.2014.07.007

[5] Chen L, Qiu Q, Jiang Y, Wang K, et al. Large-scale ruminant genome sequencing provides insights into their evolution and distinct traits. Science. 2019;364(6446):eaav6202. 31221828 10.1126/science.aav6202

[6] Feleke M, Bennett S, Chen J, et al. New physiological insights into the phenomena of deer antler: a unique model for skeletal tissue regeneration. J Orthop Transl. 2020;27:57–66. 10.1016/j.jot.2020.10.012 PMC7773678 33437638

[7] Zhang R, Dong Y, Xing X. Comprehensive transcriptome analysis of sika deer antler using PacBio and Illumina sequencing. Sci Rep. 2022;12(1):16161. 36171236 10.1038/s41598-022-20244-1 PMC9519574

[8] Ba H, Wang X, Wang D, et al. Single-cell transcriptome reveals core cell populations and androgen-RXFP2 axis involved in deer antler full regeneration. Cell Rege (London, England). 2022;11(1):43. 10.1186/s13619-022-00153-4 PMC9772379 36542206

[9] Wang Q, Han R, Xing H, Li H. A consensus genome of sika deer (Cervus nippon) and transcriptome analysis provided novel insights on the regulation mechanism of transcript factor in antler development. BMC Genomics. 2024;25(1):617. 38890595 10.1186/s12864-024-10522-9 PMC11186158

[10] Wells JN, Feschotte C. A field guide to eukaryotic transposable elements. Annu Rev Genet. 2020;54:539–61. 32955944 10.1146/annurev-genet-040620-022145 PMC8293684

[11] Ibrahim MA, Al-Shomrani BM, et al. Comparative analysis of transposable elements provides insights into genome evolution in the genus *Camelus*. BMC Genomics. 2021;22(1):842. 34800971 10.1186/s12864-021-08117-9 PMC8605555

[12] Nishihara H. Retrotransposons spread potential cis-regulatory elements during mammary gland evolution. Nucleic Acids Res. 2019;47(22):11551–62. 31642473 10.1093/nar/gkz1003 PMC7145552

[13] Hua-Van A, Le Rouzic A, et al. The struggle for life of the genome’s selfish architects. Biol Direct. 2011;6:19. 21414203 10.1186/1745-6150-6-19 PMC3072357

[14] Tang Y, Ma X, Zhao S, et al. Identification of an active miniature inverted-repeat transposable element mJing in rice. Plant J. 2019;98(4):639–53. 30689248 10.1111/tpj.14260 PMC6850418

[15] Pourrajab F, Hekmatimoghaddam S. Transposable elements, contributors in the evolution of organisms (from an arms race to a source of raw materials). Heliyon. 2021;7(1):e06029. 33532648 10.1016/j.heliyon.2021.e06029 PMC7829209

[16] Diehl AG, Ouyang N, Boyle AP. Transposable elements contribute to cell and species-specific chromatin looping and gene regulation in mammalian genomes. Nat Commun. 2020;11(1):1796. 32286261 10.1038/s41467-020-15520-5 PMC7156512

[17] Zhao P, Peng C, Fang L, Wang Z, Liu GE. Taming transposable elements in livestock and poultry: a review of their roles and applications. Genet Sel Evol. 2023;55(1):50. 37479995 10.1186/s12711-023-00821-2 PMC10362595

[18] Casanova M, Moscatelli M, Chauvière LÉ, et al. A primate-specific retroviral enhancer wires the XACT lncRNA into the core pluripotency network in humans. Nat Commun. 2019;10(1):5652. 31827084 10.1038/s41467-019-13551-1 PMC6906429

[19] Yao B, Wang C, Zhou Z, et al. Comparative transcriptome analysis of the main beam and brow tine of sika deer antler provides insights into the molecular control of rapid antler growth. Cell Mol Biol Lett. 2020;2020(25):42. 10.1186/s11658-020-00234-9 PMC7487962 32944020

[20] Feschotte C, Pritham EJ. DNA transposons and the evolution of eukaryotic genomes. Annu Rev Genet. 2007;41:331–68. 18076328 10.1146/annurev.genet.40.110405.090448 PMC2167627

[21] Feschotte C. Transposable elements and the evolution of regulatory networks. Nat Rev Genet. 2008;9(5):397–405. 18368054 10.1038/nrg2337 PMC2596197

[22] Wei L, Cao X. The effect of transposable elements on phenotypic variation: insights from plants to humans. Sci China Life Sci. 2016;59(1):24–37. 26753674 10.1007/s11427-015-4993-2

[23] Chunyi L, Yan L, Wenying DYS. Deer antlers: the fastest growing tissue with least cancer occurrence. Cell Death Differ. 2023;30(12):2452–61. 37864097 10.1038/s41418-023-01231-z PMC10733395

[24] Ali A, Han K, Liang P. Role of transposable elements in gene regulation in the human genome. Life (Basel). 2021;11(2):118. 33557056 10.3390/life11020118 PMC7913837

[25] Gozashti L, Feschotte C, Hoekstra HE. Transposable element interactions shape the ecology of the deer mouse genome. Mol Biol Evol. 2023;40(4):msad069. 36947073 10.1093/molbev/msad069 PMC10089650

[26] Li Z, Xu Z, Zhu L, et al. High-quality sika deer omics data and integrative analysis reveal genic and cellular regulation of antler regeneration. Genome Res. 2025;35(1):188–201. 39542648 10.1101/gr.279448.124 PMC11789637

[27] Kunarso G, Chia NY, Jeyakani J, et al. Transposable elements have rewired the core regulatory network of human embryonic stem cells. Nat Genet. 2010;42(7):631–4. 20526341 10.1038/ng.600

[28] Kapusta A, Feschotte C. Volatile evolution of long noncoding RNA repertoires: mechanisms and biological implications. Trends Genet. 2014;30(10):439–52. 25218058 10.1016/j.tig.2014.08.004 PMC4464757

[29] Gasparotto E, Burattin FV, Di Gioia V, et al. Transposable elements co-option in genome evolution and gene regulation. Int J Mol Sci. 2023;24(3):2610. 36768929 10.3390/ijms24032610 PMC9917352

[30] Komori T. Runx2, an inducer of osteoblast and chondrocyte differentiation. Histochem Cell Biol. 2018;149(4):313–23. 29356961 10.1007/s00418-018-1640-6

[31] Li C, Suttie JM. Deer antlerogenic periosteum: a piece of postnatally retained embryonic tissue? Anatom Embryol (Berl). 2004;204(5):375–88. 10.1007/s004290100204 11789985

[32] Zhang L, Liu S, He J, et al. Identification of MORF4L1 as an endogenous substrate of CRBN and its potential role as a therapeutic target in cancer. Sci Rep. 2025;15(1):2384. 39827217 10.1038/s41598-024-82941-3 PMC11742918

[33] Ohtani H, Iwasaki YW. Rewiring of chromatin state and gene expression by transposable elements. Dev Growth Differ. 2021;63(4–5):262–73. 34050925 10.1111/dgd.12735

[34] Poss KD. Advances in understanding tissue regenerative capacity and mechanisms in animals. Nat Rev Genet. 2010;11(10):710–22. 20838411 10.1038/nrg2879 PMC3069856

[35] Lynch VJ, Leclerc RD, May G, Wagner GP. Transposon-mediated rewiring of gene regulatory networks contributed to the evolution of pregnancy in mammals. Nat Genet. 2011;43(11):1154–9. 21946353 10.1038/ng.917

[36] Emera D, Wagner GP. Transformation of a transposon into a derived prolactin promoter with function during human pregnancy. Proc Natl Acad Sci U S A. 2012;109(28):11246–51. 22733751 10.1073/pnas.1118566109 PMC3396485

[37] Baril T, Galbraith J, Hayward A. Earl grey: a fully automated user-friendly transposable element annotation and analysis pipeline. Mol Biol Evol. 2024;41(4):msae068. 38577785 10.1093/molbev/msae068 PMC11003543

[38] Yang Z. PAML 4: phylogenetic analysis by maximum likelihood. Mol Biol Evol. 2007;24(8):1586–91. 17483113 10.1093/molbev/msm088

[39] Takahata N, Kimura M. A model of evolutionary base substitutions and its application with special reference to rapid change of pseudogenes. Genetics. 1981;98(3):641–57. 7333455 10.1093/genetics/98.3.641 PMC1214464

[40] Han R, Han L, Wang S, Li H. Whole transcriptome analysis of mesenchyme tissue in Sika deer antler revealed the CeRNAs regulatory network associated with antler development. Front Genet. 2020;10:1403. 32133026 10.3389/fgene.2019.01403 PMC7040488

[41] Kong Y, Rose CM, Cass AA, et al. Transposable element expression in tumors is associated with immune infiltration and increased antigenicity. Nat Commun. 2019;10(1):5228. 31745090 10.1038/s41467-019-13035-2 PMC6864081

[42] da Huang W, Sherman BT, Lempicki RA. Bioinformatics enrichment tools: paths toward the comprehensive functional analysis of large gene lists. Nucleic Acids Res. 2009;37(1):1–13. 19033363 10.1093/nar/gkn923 PMC2615629

[43] She X, Rohl CA, Castle JC, et al. Definition, conservation and epigenetics of housekeeping and tissue-enriched genes. BMC Genomics. 2009;10:269. 19534766 10.1186/1471-2164-10-269 PMC2706266

[44] Kim D, Paggi JM, Park C, Bennett C, Salzberg SL. Graph-based genome alignment and genotyping with HISAT2 and HISAT-genotype. Nat Biotechnol. 2019;37(8):907–15. 31375807 10.1038/s41587-019-0201-4 PMC7605509

[45] Anders S, Pyl PT, Huber W. HTSeq-a Python framework to work with high-throughput sequencing data. Bioinformatics. 2015;31(2):166–9. 25260700 10.1093/bioinformatics/btu638 PMC4287950

